# Factors in the disaster mitigation process for micro and small culinary enterprises in Indonesia

**DOI:** 10.4102/jamba.v15i1.1503

**Published:** 2023-12-23

**Authors:** Muzakar Isa

**Affiliations:** 1 Department of Management, Faculty of Economics and Business, Muhammadiyah University of Surakarta, Surakarta, Indonesia

**Keywords:** COVID-19, culinary businesses, disaster risk, disaster mitigation, vulnerability, exposure, sensitivity, adaptive capacity

## Abstract

Coronavirus disease 2019 (COVID-19) has predisposed most business activities, including the culinary business. The higher the vulnerability rating of a business, the more significant the risk. This study aims to analyse the vulnerability of businesses based on the dimensions of exposure, sensitivity and adaptive capacity to determine priority factors in disaster risk mitigation in order to maintain business continuity. This research was conducted in Surakarta City, Central Java province, Indonesia. The sample was selected using purposive random sampling based on business experience of at least two years, and were not hawkers. Data collection was carried out through structured interviews. This study utilised a qualitative approach using an index. The vulnerability model was developed to assess the vulnerability of culinary businesses to COVID-19. COVID-19 is a threat in the health industry from the macro external environment of business. It is considered the exposure dimension. The sensitivity dimension consists of business characteristics, business owner-manager demographics, and product and supplier characteristics. The demographic dimension of the business owner-manager has high vulnerability and has a high contribution to the sensitivity variable as the most vulnerable variable. The adaptive capacity dimension consists of human capital, economic capital, institutional capital, managerial capital, and supply chain capital. Managerial competencies are an adaptive capacity dimension that has a high vulnerability. The vulnerability of business to the threat of the COVID-19 pandemic is a model for culinary business managers and owners in determining priority factors in disaster risk mitigation in order to maintain business continuity.

**Contribution:**

This study analysed the vulnerability of culinary businesses for micro and small businesses. Low vulnerability means high resilience. Business resilience is conceptualised as a production function that is predisposed by various combinations of inputs from exposure, sensitivity, and adaptive capacity variables.

## Introduction

Coronavirus disease 2019 (COVID-19) has majorly disrupted the economic field by pushing many micro and small enterprises (MSEs) into failure (Giones et al. 2020). Culinary businesses are MSEs that have low resilience or a high level of vulnerability to the threat of uncertainty in the business environment (Isa et al. 2021; Wieczorek-Kosmala, Monika & Anna Doś 2021). The position of the culinary business is not in line with its crucial role in job creation and increasing people’s income (Davlasheridze & Geylani 2017; Purnomo et al. 2021), regional economic growth (Goel 2022; Praswati et al. 2021), poverty alleviation (Nursini 2020), and strengthening food security (Hadi 2020). There are 90% of national food products provided by MSEs (Hadi 2020). Micro and small enterprises play a vital role in supporting Indonesia’s economic growth in which their number has approximately 99.99% of the total existing business actors, provide around 117 million jobs, and contribute 60% to the National gross domestic product (GDP) (Badan Pusat Statistika [BPS] 2022).

Culinary businesses are MSEs that faced the highest risk of the threat of COVID-19 (Messabia, Fomi & Kooli 2022), where many have experienced a decrease in income, and some have gone bankrupt (Setiyawan & Isa 2022). This high risk is presumably caused by the high vulnerability of the business (Kamalipoor et al. 2022). Analysis of business vulnerabilities is one of the leading pillars for formulating a business strategy (Cowan & Wright 2016). The vulnerability of business to the COVID-19 pandemic is an issue of study to investigate (Isa, Sugiyanto & Susilowati 2018), which is driven by increased business vulnerability to internal and external factors (Eltantawy 2016). The COVID-19 pandemic has severely affected various aspects of the economy and business (Rahmayani, Oktavilia & Putri 2021), including the health level of the employee, decreased performance for shipments from suppliers and to consumers, and changes in consumer behaviour.

Most countries in the world, including Indonesia, reduced the spread of the COVID-19 virus by limiting human activity, which has an undesirable impact on economic and business activities (Sharma et al. 2020), where Indonesia’s economic growth in 2020 was 2.97% and decreased from the previous year, namely 5.02%. Surakarta as a culinary business centre in Indonesia (Rahadhini, Choerudin & Dwi, 2021) has experienced a negative impact from the COVID-19 pandemic; many of them have experienced losses and even closed their businesses. The accommodation, food, and beverage industry for Surakarta City in 2020 dropped to –16.20%.

The vulnerability of culinary businesses to the threat of COVID-19 is conceptualised as a production function, where vulnerability is interpreted as the output of MSEs, which is predisposed by various combinations of inputs (Gaffar et al. 2022; Isa & Mangifera 2019). The absence of data on the performance of culinary MSEs from non-financial aspects, such as the level of business vulnerability to threats from changes in the external environment, is a research problem. In addition, no data were revealed on what input variables have a significant influence on the vulnerability of business. Therefore, this issue is a topic worth looking into to a greater extent to reduce the vulnerability of MSEs to maintain the sustainability of business in this uncertain era.

Culinary MSEs have enormous challenges, that is changes in the business environment that are rapid and full of uncertainties (Gaffar et al. 2022; Otengei & Ahebwa 2021). Consequently, they are required to respond periodically and continuously to changes in their external environment and evaluate their internal capabilities (Otengei & Ahebwa 2021). Business managers must continuously emphasise the uncertainty of the business environment, both internal and external, in managing their business. Not many culinary businesses can survive in an uncertain business environment (Day & Schoemaker 2016). Thus, it is necessary to identify and analyse the level of vulnerability of culinary businesses and the input factors that affect this level of vulnerability so that they can be used as a basis for business managers and the government to determine priority factors in disaster risk mitigation in order to maintain business continuity.

## Theoretical framework

The scale of the culinary business risk because of the COVID-19 pandemic is influenced by the level of vulnerability of the business (Kamalipoor et al. 2022). The higher the level of vulnerability of the business, the higher the risk when there is a disaster or disruption (Isa et al. 2021). Isa and Mardalis (2022) define vulnerability as the lack of ability of a business to deal with uncertainty. Kamalipoor (2022) states that vulnerability is the tendency for a business to be affected by a hazard or potential for change. Aleksic et al. (2014); Lo et al. (2019); Isa et al. (2018), and Kamalipoor (2022) describe vulnerability as a three-dimensional system, namely exposure: the extent to which the system is exposed to hazards, sensitivity: the extent to which a system is affected, adaptive capacity: the ability of the system to adapt to change.

The COVID-19 pandemic has instigated many culinary businesses to suffer losses and many have even closed their businesses. The results of the BPS survey revealed that 92.47% of culinary business experienced a decrease in income because of the COVID-19 pandemic (BPS 2021). The COVID-19 pandemic has triggered a number of supply chain bottlenecks that have delayed shipment of raw materials and demand to decline (Eggers 2020; Seetharaman 2020); thus, many of them reduced the need for employees (Nicola 2020). The number of employees exposed to COVID-19, the frequency, severity, and duration of exposure, and the location of the business are exposure factors that determine the level of vulnerability of a business (Khan et al. 2022; Wang, Viseu Cardoso and Forgaci 2022; Yang 2020).

The sensitivity of the culinary business is influenced by the dimensions of business characteristics, demographics of business owners, and characteristics of the supply chain and supply. The business character dimension influences the ability of the business to deal with COVID-19 (Kamalipoor 2022). The size of the business is a critical business characteristic (Davlasheridze & Geylani 2017; Eggers 2020). Micro and small enterprises have closer relationships with suppliers and customers so they have access to valuable information. During a pandemic, they face resource scarcity and decreased demand, so they are more vulnerable to environmental changes. The smaller the size of the business, the more vulnerable it is to the COVID-19 pandemic. Eggers (2020) considers new businesses to be more vulnerable than old businesses because of a lack of experience and network. In addition, business characteristics that affect sensitivity include the number of business branch offices (Song et al. 2016), previous business performance (Sydnor et al. 2017), ownership of business legality (Sydnor et al, 2017), and financial resources (Khan & Sayem 2013).

The sensitivity of the culinary business is also influenced by the demographics of the owner or manager, namely gender (Lo et al. 2019; Song et al. 2016; Sydnor et al. 2017), level of education (Lo et al. 2019; Orhan 2016; Sydnor et al. 2017), business experience (Sydnor et al. 2017), competency in managing a business (Isa & Mardalis 2022), and experience dealing with previous disasters (Orhan 2016). Women and entrepreneurs have a negative relationship (Zapata-Huamaní et al. 2019). Women business owners prefer to be safe when facing a pandemic, such as closing their business and reopening after normal conditions. Owners with higher education prefer to continue their business and not close the business compared with owners with low educational backgrounds (Sydnor et al. 2017). Educated and knowledgeable managers and/or owners have more quality and efficiency in transferring knowledge to reduce the vulnerability of business. Experience in dealing with previous disasters can increase the willingness and ability to mitigate and adapt (Isa, Sugiyanto & Susilowati2015; Orhan 2016).

In addition to the dimensions of business characteristics and demographics of owners or managers, the dimensions of the supplier, product, and market characteristics also affect the sensitivity. The COVID-19 pandemic disrupted the distribution of products from suppliers and to consumers, which caused huge losses for MSEs (Isa et al. 2021; Stecke & Kumar 2009). Types of products; services; price; and availability of raw materials are key business aspects that play a role in determining the level of vulnerability (Orhan 2016; Khan & Sayem 2013).

Adaptive capacity is a system for better adapting to external changes (Isa et al. 2021). Low adaptive capacity shows an indicator of high business vulnerability to natural disasters (Davlasheridze & Geylani 2017). Adaptive capacity plays a crucial role in the final susceptibility of culinary ventures because it determines massive effect scales. Various resources, including human, institutional, management, financial, and supply chain capital affect vulnerability (Marshall & Schrank 2014). Human capital is an intangible organisational asset and is referred to unique and knowledgeable people in the organisation (Johannesson & Jorgensen 2017). Human capital can be interpreted as the explicit knowledge of people in culinary business such as knowledge, experience, learning ability, or knowledge creation capability (Delgado-verde, Castro & Amores-salvado 2016). Human capital has several indicators, that is employee capabilities, satisfaction, and endurance. Academic education, public information, and specialisation are significant characteristics of human capital.

Institutional capacity is represented by governance structures and disaster risk reduction systems (Ludena, Sanchez-aragon & Miller 2015) as a component of effective adaptive capacity in reducing vulnerability (Ludena et al. 2015). Institutions are fundamental determinants of economic and exchange behaviour (Isa & Mangifera 2019), and influence innovation (Arabiyat et al. 2019). Isa and Mangifera (2019) explain that humans develop institutions to discipline exchanges and reduce uncertainty. Institutions play a central role during the COVID-19 pandemic to respond to high uncertainty. Furthermore, managerial action becomes a determinant of adaptive capacity (Polsky, Neff & Yarnal 2007). Culinary business strategic plans prominently affect business vulnerability. Risk management should be an integral part of the strategic planning process. Owners or managers of culinary businesses need to integrate crisis management into their strategic planning to reduce vulnerability to the COVID-19 pandemic (Alvarez, Afuah & Gibson 2018). Entrepreneurs have to make decisions under uncertain conditions because the environment is full of uncertainties. Uncertainty affects entrepreneurs in decision-making (Alvarez et al. 2018).

The COVID-19 pandemic presents an enormous challenge to the culinary business, namely how can they survive by reducing physical contact with suppliers and consumers (Seetharaman 2020). Culinary businesses must change their business model according to environmental conditions and consider all aspects of the resources they have. The availability of financial resources is a vital component for investing in developing a new system. Innovation for suppliers and consumers requires financial resources (Eggers 2020). Financial capital is a critical factor in dealing with COVID-19. In addition to financial capital, supply chain capability is a highly significant aspect for culinary businesses to reduce vulnerability or increase business resilience to the COVID-19 pandemic (Ekanayake et al. 2020; Susilowati et al. 2018). The COVID-19 pandemic disrupted numerous supply chains (Kirk & Rifkin 2020; Sharma et al. 2020).

## Research methods and design

This research was conducted in Surakarta City, Central Java province, Indonesia. The city of Surakarta is one of the culinary destination centres in Indonesia, where this city has 716 culinary businesses with 1729 types of menus (BPS 2022). During the COVID-19 pandemic, as a regional superior product, the culinary business experienced an undesirable impact. The food and beverage industry in Surakarta City has a negative growth of –16.20%. The 716 businesses constitute the population and will be selected by purposive random sampling based on at least 2 years of business experience, experience in dealing with disasters, namely COVID-19, and are not hawkers. Data collection was completed through structured interviews supported with questionnaires and Focus Group Discussions (FGD) with relevant stakeholders (Creswell & Creswell 2018).

The vulnerability index is compiled based on three variables, including exposure, sensitivity, and adaptive capacity. The three variables are elucidated into several dimensions and indicators (Table 1).

**TABLE 1:** Variables, dimensions, and indicators of vulnerability.

Variable	Dimensions	Indicator
Exposure	Coronavirus Threat	Number of employees exposed to COVID-19
		The average number of days an employee is exposed to COVID-19
		Zone categories for business locations
		Frequency of employees exposed to COVID-19
		The number of employees with comorbidities
Sensitivity	Business characteristics	Size of business (micro, small, and medium)
		Business age (length of business)
		Sales before COVID-19
		Legal ownership
		Source of capital
	Owner and/or manager demographics	Gender
		Level of education
		Business experience
		Previous disaster experiences
	Supplier, product, and market characteristics	Availability of suppliers
		Availability of raw materials
		Raw material prices
		Selling price
		Market reach
Adaptive capacity	Human capital	Employee availability
		Willingness and ability to change and take risks
		Knowledge of how to serve the market, and customer problems
	Economic capital	Type of income source
		Previous sales level
		Allocation of funds for risk mitigation
	Institutional capital	Government assistance
		Ease of starting a business
		Ease of obtaining business licenses
	Managerial capital	Ability to make business plans
		Ability to make decisions in uncertain conditions
		Ability to direct business
	Supply chain capital	Availability of product information
		Price affordability
		Dependence on one partner
		Changes in consumer behaviour

*Source*: Kamalipoor (2022); Kamalipoor, M., Akbari, M., Hejazi, S.R. & Nazarian, A., 2022, ‘The vulnerability of technology-based business during COVID-19: An indicator-based conceptual framework’, *Journal of Business & Industrial Marketing* 38(5), 983–999. https://doi.org/10.1108/JBIM-10-2020-0455; Sahu, N. & Mishra, M.M., 2021, ‘Assessing the vulnerability index of COVID-19 pandemic in India’, *Geography, Environment, Sustainability* 14(4), 131–139. https://doi.org/10.24057/2071-9388-2021-059

COVID-19, coronavirus disease 2019.

This study used index analysis. The vulnerability index of culinary businesses to the threat of COVID-19 was determined by compiling all scores for each indicator of vulnerability to the threat of COVID-19, which consisted of exposure, sensitivity, and adaptive capacity (Isa et al. 2021; Kamalipoor et al. 2022). The weighting of each variable was completed by considering the influence of each aspect in forming the vulnerability aspect. The more significant the influence of an aspect, the higher the weight. Weighting is obtained through FGD with stakeholders. The vulnerability index was determined by multiplying the total score of all indicators by the weight of exposure, sensitivity, and adaptive capacity (Weis et al. 2016). The MSEs vulnerability index is expressed in the following formula (Isa et al. 2021):

$$\text{V}=\sum_{\text{i}=1}^{3}\left({\text{W}}_{1}{\text{X}}_{1})+({\text{W}}_{2}{\text{X}}_{2})+({\text{W}}_{3}{\text{X}}_{3}\right)$$ [Eqn 1]

where V is the culinary business vulnerability index to COVID-19, W1: the exposure variable weight, X1: the exposure variable score, W2: the sensitivity variable weight, X2: the sensitivity variable score, W3: the adaptive capacity variable weight, X4: adaptive capacity variable score. The results of the vulnerability index were grouped into five groups, namely very low, low, moderate, high, and very high.

### Ethical considerations

This article followed all ethical standards for research without direct contact with human or animal subjects.

## Results and discussion

The COVID-19 pandemic is an emergency for the health industry. Policies for handling the COVID-19 pandemic have had an impact on various economic and business activities. Countries and regions that apply stricter social restrictions will tend to experience more significant contractions. Social restrictions caused people’s mobility to decrease sharply, which then impacted economic activity. The COVID-19 pandemic has had an impact on all economies, both households and business owners. Households experience a decrease in income because of a reduction in the number of working days or layoffs. The business owners experienced a decline in sales, decreased revenue, and experienced bankruptcy.

The food and beverage industry is part of the manufacturing industry, the creative economy, and the tourism industry in Surakarta City (Nasir, Irmawati & Isa 2021), which have been heavily affected by the COVID-19 pandemic. Micro and small food and beverage businesses in Surakarta City are the leading regional businesses that experienced the most severe impact during the COVID-19 pandemic. The decline in their performance was caused by disruptions in the supply of raw materials, decreased production capacity utilisation during the pandemic, and reduced demand. The performance and sustainability of the business are influenced by the level of vulnerability of the business to the threat of COVID-19, where the higher the level of vulnerability of the business, the lower the performance.

Kamalipoor et al. (2022) mentioned that the level of vulnerability of a business to the threat of COVID-19 affects the magnitude of the risk of a business. High risk has a positive effect on decreasing people’s income (Davlasheridze & Geylani 2017; Purnomo et al. 2021), increasing the amount of poverty (Nursini 2020), and regional economic growth rate (Goel 2022; Isa et al. 2015). The high risk indicates an unresolved economic problem, namely inefficiency in the management of business (Planinc et al. 2022). Risk depicts the ability of a business to face the threat of COVID-19 (Isa et al. 2021). Low risk describes a high level of business resilience to the threat of COVID-19.

The level of vulnerability or the level of resilience of a business is affected by exposure, sensitivity, and adaptive capacity (Aleksic et al. 2014; Isa et al. 2018; Lo et al. 2019; and Kamalipoor 2022). Vulnerability assessment is significantly conducted to determine the level of vulnerability of business in an area and determine aspects of the causes so that it becomes material to policymaking for the government and business managers. Exposure is one of the three determinants of business vulnerability to the threat of COVID-19. Weis et al. (2016) and Kamalipoor (2022) emphasised that exposure is an aspect of vulnerability that explains the extent to which the threat of COVID-19 affects business risks related to the number of employees exposed to COVID-19, the average number of days employees are exposed to COVID-19, the category of the COVID-19 zone for the location of the business, the frequency of employees exposed to COVID-19, and the number of employees who are co-workers.

Table 2 portrays the indicators for the dimensions of the coronavirus threat. This dimension is explicated by five indicators, namely the number of employees exposed to COVID-19, the average number of days employees are exposed to COVID-19, the category of business location zone, and the frequency of employees exposed to COVID-19, which is in the moderate vulnerability category. One indicator, namely the number of co-workers, is in the low vulnerability category and one indicator is in the high category, namely the zone where the food and beverage business is located. During the pandemic, many culinary business centres were in the red zone or high-risk zone. There were five zone groups during the COVID-19 pandemic, namely high-risk zone, moderate-risk zone, low-risk zone, and unaffected zone: no positive cases of COVID-19 were recorded and no case zone. Culinary businesses that are in the red zone needed to close their businesses and were directly impacted. The social restriction policy in this zone is strictly enforced and causes people’s mobility to decrease sharply resulting in a negative impact on various economic activities.

**TABLE 2:** Level of vulnerability of business based on exposure aspects.

No.	Dimensions	Indicator	Vulnerability score	Category
1	Coronavirus Threat (0.46)	Number of employees exposed to COVID-19	0.38	Low
2	Coronavirus Threat (0.46)	The average number of days an employee is exposed to COVID-19	0.55	Moderate
3	Coronavirus Threat (0.46)	Zone categories for business locations	0.63	High
4	Coronavirus Threat (0.46)	Frequency of employees exposed to COVID-19	0.51	Moderate
5	Coronavirus Threat (0.46)	Number of employees with comorbidities	0.21	Low

COVID-19, coronavirus disease 2019.

Sensitivity is the second aspect of vulnerability, which represents the condition of the owner or manager of a business in dealing with the threat of COVID-19 (Kamalipoor et al. 2022). Table 3 shows the sensitivity index composed of three dimensions, namely business characteristics, owner and/or manager demographics, and supplier, product, and market characteristics. The characteristic business dimension consists of five indicators, namely size of business, business age, pre-COVID-19 sales, legal ownership, and sources of capital. This dimension has four indicators that are in the moderate category, namely size of business, pre-COVID-19 sales, legal ownership, and sources of capital, and one indicator that is in the high category, namely the age of the business. Furthermore, the owner and/or manager demographics dimension consists of four indicators, namely gender, education level, business experience, and previous disaster experience, where all these indicators are in the high category. The supplier, product, and market characteristics dimensions consist of four indicators, namely supplier availability, raw material availability, raw material prices, selling prices, and market reach, where all indicators are in the moderate category. These results show that the owner and/or manager demographics dimension has the greatest influence in forming the sensitivity index and is the most vulnerable dimension for business to the threat and risk of COVID-19.

**TABLE 3:** Level of vulnerability of business based on sensitivity aspect.

No.	Dimension	Indicator	Vulnerability score	Category
1	Business characteristics (0.49)	Size of business	0.51	Moderate
		Business age	0.64	High
		Pre-COVID-19 sales	0.37	Low
		Legal ownership	0.53	Moderate
		Source of capital	0.42	Moderate
2	Owner and/or manager demographics (0.68)	Gender	0.74	High
		Level of education	0.71	High
		Business experience	0.65	High
		Previous disaster experiences	0.63	High
3	Supplier, product, and market characteristics (0.50)	Availability of suppliers	0.44	Moderate
		Availability of raw materials	0.41	Moderate
		Raw material prices	0.55	Moderate
		Selling price	0.52	Moderate
		Market reach	0.58	Moderate

COVID-19, coronavirus disease 2019.

Adaptive capacity is the third variable in shaping the vulnerability of business to the threat of COVID-19. Isa et al. (2015) expressed adaptive capacity as an aspect of vulnerability that explains the ability of business to reduce risk from the threat of COVID-19. The adaptive capacity index is composed of five dimensions, namely human capital, economic capital, institutional capital, managerial capital, and supply chain capital. Each dimension has 3–4 indicators.

The first dimension of the adaptive capacity variable is human capital, which has three indicators, namely the availability of employees, willingness and capability to change and take risks, and knowledge about how to serve the market and customer problems. All indicators on this dimension have a moderate category. The second dimension is economic capital, which has three indicators, involving the type of source of income, the level of pre-COVID sales, and the allocation of funds for risk mitigation. The two indicators on this dimension are in the moderate category, namely the type of income source, and the level of pre-COVID-19 sales, while the indicator for allocating funds for risk mitigation is in the high category.

The third dimension is institutional capital. This dimension has three indicators, namely government assistance, ease of starting a business, and ease of obtaining business licenses, where all indicators in this dimension are in the moderate category. Furthermore, the fourth dimension is managerial capital. This dimension has three indicators, including the ability to make business plans, the ability to take decisions in conditions of uncertainty, and the ability to direct business, where all indicators on this dimension have a high category.

The last dimension is supply chain capital. This dimension has four indicators, that is the availability of product information, price affordability, dependence on one partner, and changes in consumer behaviour. Two indicators, the availability of product information and price affordability, are in the high category while the other two indicators are in the moderate category.

The index of the vulnerability of food and beverage businesses in Surakarta City to the threat of COVID-19 is compiled based on the scores of all indicators of the aspects of exposure, sensitivity, and adaptive capacity as discussed earlier. This score is then multiplied by the weight of each aspect of exposure, sensitivity, and adaptive capacity. The weight of vulnerability forming aspects was obtained from FGDs with stakeholders and obtained exposure weights of 30%, sensitivity of 35%, and adaptive capacity of 35%. The results of the calculation of the vulnerability index (Table 4) yielded a vulnerability index score for the food and beverage business of 0.52, which is in the moderate category.

**TABLE 4:** Vulnerability index of food and beverage businesses to the threat of coronavirus disease 2019.

Description	Exposure		Sensitivity		Adaptive capacity		Index vulnerability	
	Score	Weight	Score	Weight	Score	Weight	Score	Information
Vulnerability Index	0.46	0.30	0.56	0.35	0.52	0.35	0.52	Moderate
	0.14		0.20		0.18			

The food and beverage businesses in Surakarta City had a moderate level (0.52) of vulnerability during the COVID-19 pandemic, lying in the range of 0.40–0.60. The higher the level of vulnerability of a business, the more significant the risk (Isa & Mardalis 2022; Li et al. 2023), so various strategies and programmes from the government and business managers are needed to reduce the level of vulnerability of business. The index scores for each indicator in Table 2, Table 3 and Table 5 are significant materials for strategy formulation for the business field and policy formulation for the government. Strengthening the vulnerability of the culinary business is essential to increase people’s income, reduce unemployment and poverty, and achieve a set level of economic growth.

**TABLE 5:** Level of vulnerability of business based on adaptive capacity aspects.

No.	Dimension	Indicator	Vulnerability score	Category
1	Human capital (0.47)	a. Employee availability	0.43	Moderate
		b. Willingness and ability to change and take risks	044	Moderate
		c. Knowledge of how to serve the market and customer problems	0.54	Moderate
2	Economic capital (0.58)	a. Type of source of income	0.54	Moderate
		b. Pre-COVID-19 sales levels	0.51	Moderate
		c. Allocation of funds for risk mitigation	0.69	High
3	Institutional capital (0.48)	a. Government assistance	0.59	Moderate
		b. Ease of starting a business	0.40	Moderate
		c. Ease of obtaining business licenses	0.45	Moderate
4	Managerial capital (0.67)	a. Ability to make business plans	0.64	High
		b. Ability to make decisions in uncertain conditions	0.68	High
		c. Ability to direct business	0.76	High
5	Supply chain capital (0.60)	a. Availability of product information	0.69	High
		b. Price affordability	0.61	High
		c. Dependence on one partner	0.59	Moderate
		d. Changes in consumer behaviour	0.53	Moderate

Sensitivity of business is the aspect that has the highest influence as a business vulnerability index, followed by adaptive capacity and exposure. This finding supports previous research, which uncovered that sensitivity is the most vulnerable aspect. Owner and/or manager demographics are a sensitivity dimension that has the highest indicator score, causing sensitivity to be the most vulnerable aspect, compared with exposure and adaptive capacity. The owner and/or manager demographics indicator explains that many culinary business managers are women with high school education levels. Many of them have been in business for less than 5 years, and they have no previous experience in dealing with disturbances or disasters. Dimensions and indicators that have a high vulnerability score must be followed up immediately to maintain business continuity.

Economic capital, managerial capital, and supply chain capital are three dimensions of adaptive capacity with high indicator scores. It is imperative that these three elements be strengthened right away because they are a key aspect of the success or failure of a business. Businesses managers must respond to increase the output of business. Analysis of the vulnerability of small culinary businesses to COVID-19 is urgent for businesses and the government. The study’s findings provide a solid basis for maintaining the survival of the culinary business and sustainable economic development policies (Davlasheridze & Geylani 2017).

## Conclusion

Analysis of the vulnerability of culinary businesses to the threat of COVID-19 is significant as a foundation for maintaining business continuity and sustainable economic development policies. This vulnerability analysis was completed based on three aspects, including exposure, sensitivity, and adaptive capacity. A low level of vulnerability is obtained if business managers understand the characteristics and components of a disaster threat (COVID-19). In addition, the government and business managers must recognise business characteristics, job characteristics, demography of owners and/or managers, and characteristics of suppliers and/or products as vital components for reducing the sensitivity index. Hence, business managers must be able to determine how far they are prepared to face crises at various levels, including resources, technological capital, human capital, social capital, economic capital, institutional capital, managerial capital, and supply chains. Then, they must identify their weak points and deficiencies to respond adaptively and effectively. These are effective ways they can choose to survive and reduce their vulnerability.

The vulnerability level of the culinary business in Surakarta City is 0.52 and is in the moderate category. Sensitivity of business is the most vulnerable of vulnerability index-forming variable, followed by adaptive capacity and exposure. Furthermore, owner and/or manager demographics is a sensitivity dimension that has the highest vulnerability score. This research is beneficial for culinary business owners and managers that can be applied to crises and disasters. Owners and managers can develop strategies for dealing with shocks and disasters, that is various possible internal and external incidents, and they can prepare for crises.

One of the limitations of this research that needs attention in future research is the limited data in describing all micro and small businesses. Therefore, it is recommended for further research to analyse various types of businesses and all business sizes, not only culinary MSEs.
